# Evaluating changes in hypoglossal nerve stimulator use over time and long-term adherence

**DOI:** 10.1007/s11325-025-03415-y

**Published:** 2025-07-18

**Authors:** Nicholas R. Lenze, Dania Alazawi, Meghan Dailey, Christopher Brown, Paul T. Hoff, Cathy A. Goldstein

**Affiliations:** 1 Department of Otolaryngology– Head and Neck Surgery, University of Michigan Medical School, 1500 East Medical Center Drive, Ann Arbor, MI 48108 USA; 2 Advanced Research Computing Technology Services, University of Michigan, Ann Arbor, MI USA; 3 Sleep Disorders Center, Department of Neurology, University of Michigan Medical School, Ann Arbor, MI USA

**Keywords:** Obstructive sleep apnea, Hypoglossal nerve, Electrical stimulation therapy, Adherence, Treatment outcome, Machine learning

## Abstract

**Purpose:**

Evaluate patient phenotypes and longitudinal patterns of hypoglossal nerve stimulator (HGNS) use and identify predictors of long-term HGNS adherence.

**Methods:**

Patients who underwent HGNS implantation for obstructive sleep apnea (OSA) from 2017 to 2023 and had available data through 9 months post-device activation were included. Adherence rate was defined as percentage of patients using the device for at least 4 h for 70% of nights. Repeated measures ANOVA and Cochran’s Q tests were used to analyze changes in HGNS use over time. A k-means clustering analysis was used to identify HGNS user subgroups with shared characteristics and associations with HGNS use.

**Results:**

A total of 59 patients were included, with a mean (SD) age of 62.7 (11.2) years, mean (SD) body mass index of 28.5 kg/m2 (3.2), and an average pre-operative apnea-hypopnea index (AHI) of 38.7 events/hour; the majority were male (78%) and White (98.3%). Patients used their HGNS devices on average for 81.1% of nights (SD 23.5%) and 362 min/night (SD 115), with 0.96 (SD 1.4) pauses/night at 9 months post-activation. The mean percentage of nights and the time/night used decreased significantly over the first 9 months (*p* < 0.001 for both), while pauses/night increased (*p* = 0.008). The estimated adherence rate was 52.5% at 9 months. The cluster analysis revealed subgroups with shared characteristics; however, clusters were not associated with HGNS use.

**Conclusions:**

HGNS use appears to decrease over the first nine months after activation. Additional research is warranted to investigate drivers of HGNS use decrement. Given lack of a standardized definition for adherence, future studies should report more granular HGNS use metrics to facilitate comparison across studies.

**Supplementary Information:**

The online version contains supplementary material available at 10.1007/s11325-025-03415-y.

## Introduction

Obstructive sleep apnea (OSA) affects an estimated 25% of adults in the United States and confers a significant burden of disease in terms of quality of life, cognitive function, cardiovascular mortality, and motor vehicle accidents [1, 2]. Although positive airway pressure (PAP) is the first-line treatment for moderate to severe OSA, its real-world efficacy is challenged by poor adherence, with approximately 25% of PAP users considered non-adherent within the first 90 days of use based on the US Center for Medicare and Medicaid Services (CMS) criteria (at least 4 h of use for 70% of nights) [3, 4]. Given that multiple investigations have identified that more than 6 h of PAP use per night are required for normalization of objective and subjective alertness, as well as improved cognitive function, adherence is crucial to favorable outcomes [5–7]. In the past decade, hypoglossal nerve stimulation (HGNS) has emerged as an effective alternative for select OSA patients who struggle to adhere to PAP. Over 100,000 patients have been treated with the Inspire (Golden Valley, MN) HGNS stimulator worldwide since 2014 [8]. Similar to PAP therapy, HGNS relies on consistent long term device adherence; withdrawal from HGNS therapy is associated with worse subjective sleepiness and objective outcomes [9, 10]. 

Studies of the Adherence and Outcome of Upper Airway Stimulation for OSA International Registry (ADHERE) database have found that over 90% of patients who undergo HGNS report satisfaction at long-term follow-up [11], and the mean device use is approximately 5.6 h/night at around 14 months post-activation [12]. However, HGNS adherence may vary depending on how it is defined, and few studies have used adherence definitions comparable to the CMS criteria for PAP. One study found that only 65% of patients met adherence criteria when defined as use of at least 28 h/week [13]. More granular HGNS usage metrics, tracked longitudinally, are needed to identify patterns of use.

In addition to more granular HGNS use data, a better understanding of factors associated with long-term HGNS adherence are crucial to help guide patient selection and personalize postoperative counseling. Several factors associated with HGNS use have been identified in current literature including age [14–16], body mass index (BMI) [12], apnea-hypopnea index (AHI) [16], anxiety [13], insomnia [17], and restless leg syndrome [18]. However, most of these studies have been limited to the first three months after device activation, and few have evaluated longitudinal changes in HGNS adherence. Furthermore, most of these studies have relied on regression modeling techniques which may fail to capture the intersectionality of patient and clinical factors that affect HGNS use.

To address this gap in literature, we evaluated an institutional cohort of patients who underwent HGNS implantation and had device use data up to 9 months post activation. The 9-month period was chosen to capture adherence patterns after the postoperative window where device adjustments are typically made (self-titration of the device (1–3 months), a titration sleep study (~ 3 months), and a new baseline sleep study (~ 6 months); using 9-month data rather than 12-month data also helped maximize the sample size. In addition to assessing change in HGNS use over time, we utilized unsupervised machine learning to identify distinct patient clusters. We evaluated cluster associations with HGNS use, in addition to traditional modeling techniques.

## Methods

### Patient sample

We retrospectively identified all adult patients who underwent HGNS implantation at our institution from 2017 to 2023. All patients were evaluated in a multidisciplinary sleep clinic prior to HGNS implantation. Patients were candidates for HGNS implantation if they had moderate to severe OSA (with < 25% central apneic events), were unable to tolerate PAP, and had lack of complete concentric collapse of the palate on drug-induced sleep endoscopy (DISE). All patients were implanted with the Inspire therapy device (Inspire Medical Systems, Inc., Golden Valley, MN).

Patients are seen in clinic 1 to 2 months after HGNS implantation for device activation by a sleep surgeon and an Inspire representative. There were a total of three different Inspire representatives who saw patients over the study period. Patients are given instructions on device use, such as turning the device on/off and pausing the device, as well as titrating the voltage. After activation of their device, patients are instructed to self-titrate in their home environment. The voltage starts at their functional threshold, and they are instructed to titrate up about 0.1 V per week as tolerated over the course of about 3 months with a total range of 1 V. During this 3-month period, the Inspire representatives are intermittently checking the SleepSync platform and will reach out to the sleep medicine provider if there are any concerns about patient adherence. The sleep medicine provider will then call the patient or have the patient come into clinic for adjustments to device settings if deemed necessary. At approximately 3 months post-activation the patients undergo an in-lab titration sleep study. Patients then have a clinic visit with the sleep medicine provider and Inspire representative 2–3 weeks after the titration study to discuss the results and make adjustments if necessary. Patients undergo a final non-titration sleep study (home or in-lab) about 6 months after the titration study.

Electrode configuration, pulse width, and frequency were adjusted at device activation by the Inspire representative and sleep surgeon based on tongue protrusion and patient comfort. These settings were adjusted on an as needed basis at subsequent visits. Start delay was set at a default setting of 30 min and pause time was set at a default setting of 15 min. Patients were instructed to adjust these according to their own preferences and sleep patterns. Patient control for range of voltage was set at 1 V.

Device use data was obtained via SleepSync, a secure online platform that collects and stores HGNS use data via Bluetooth from the patient’s device or direct USB upload in clinic. Although SleepSync was available at our institution, it may not be available at other institutions or outside the United States. Patients were excluded from this study if they did not have at least 9 months of HGNS use data available.

### Variables

The primary outcome variable was HGNS use, which included percentage of nights used, percentage of nights used for at least 4 h, time used per night (minutes) and pauses per night. These variables were tabulated for 0–3 months, 3–6 months, and 6–9 months post-activation. Percentage of nights used was defined as the number of nights that the device was used at all divided by the total number of nights in the time period of interest. Percentage of nights used for at least 4 h was defined as the number of nights the device was used for 4 h or more divided by the total number of nights in the time period of interest. The number of patients in each time period using the device for at least 4 h for 70% of nights was calculated from these data to estimate adherence rate. All HGNS use and voltage data were extracted from the SleepSync platform.

Electronic medical records were reviewed to collect relevant sociodemographic, anthropometric, and clinical variables. Neighborhood level income was obtained from DataDirect, a self-service data extraction tool that links electronic health record data with geocoded socioeconomic data based on patients’ home addresses. Variables were chosen based on literature review and discussion with the study team about factors that could plausibly affect HGNS use. Relevant comorbidities (e.g. insomnia, depression, anxiety, etc.) were coded as “yes” if they were included in a patient’s problem list or past medical history. Centrally acting medications included antidepressants, opioids, benzodiazepines, anxiolytics, antipsychotics, and hypnotic agents. Preoperative sleep studies included both home sleep studies and in-lab polysomnograms. Postoperative sleep studies included the most recent sleep study (e.g. performed furthest out from surgery). Apnea-hypopnea index was calculated based the American Academy of Sleep Medicine Manual for the Scoring of Sleep and Associated events, Version 3 with use of the recommended (1 A) hypopnea definition of at least a 30% reduction in airflow terminating in a 3% reduction in oxygen saturation or an arousal [19]. 

### Data analysis

Repeated measures ANOVA and Cochran’s Q tests were used to evaluate changes in HGNS use metrics over time for continuous and categorical outcome variables, respectively. Next, univariate logistic regression models were used to evaluate associations with adherence (use of the device for at least 4 h for 70% of nights). Differences in pre-operative and post-operative sleep outcomes such as Epworth Sleepiness Scale, apnea-hypopnea index, oxygen nadir, and surgical success defined by Sher_20_ criteria (> 50% reduction in AHI, and post-operative AHI < 20 events/hour) [20] were evaluated using two-sided t-tests and chi-square testing.

We next evaluated several supervised and unsupervised machine learning methods to analyze our data. We intended to focus on k-means clustering to identify groupings of data points and find associations among variables, but we also tested support vector machines, random forest, and a neural network. These supervised methods gave us insight into designing a model that could find associations between input data and expected outcomes, but a k-means clustering model was ultimately more desirable because it could potentially identify patterns among different patients; for example, patients grouped into a cluster together would potentially have similar clinical presentations, which could help predict how different patients might behave similarly. Therefore, we proceeded with a k-means clustering analysis to identify distinct subgroups associated with 9-month adherence in our dataset.

A Gaussian mixture model was then utilized to divide our data into clusters and analyze their properties. The elbow method revealed that 2–3 clusters best fit the data, and because the outcome of interest was the binary value of adherence, the final analysis contained 2 clusters. We implemented a PCA reduction to two dimensions to visualize our clusters. Three different variable groupings were tested to determine the optimal clusters (Supplemental File 1). When choosing which variables to include in clusters, we focused on pre-operative variables that could be used to potentially inform patient selection for surgery. Therefore, post-operative variables, such as stimulation amplitude, were not included in the clustering models. Grouping 1 (comprehensive) consisted of all variables listed in Table 1 except for HGNS voltage. Grouping 2 (sleep and endoscopy parameters) consisted of select variables from the pre-operative sleep study as well as drug induced sleep endoscopy (DISE) findings. Grouping 3 (a priori characteristics) included variables in our data that have been associated with HGNS use in the published peer-reviewed literature. Clustering results between the groupings were compared using the Dunn index, Pseudo F score, and Silhouette Score (Supplemental File 1). Grouping 1 (comprehensive) resulted in the most well-formed clusters, so this grouping was used to create the clusters for the subsequent analysis.

**Table 1:** Baseline characteristics

Variable	Number (%) or mean (SD)
Age, years (mean, SD)	62.7 (11.2)
Sex	
Female	13 (22.0%)
Male	46 (78.0%)
Race	
Black	1 (1.7%)
White	58 (98.3%)
Neighborhood-level household income (mean, SD)	93,296 (35,864)
Body mass index	28.5 (3.2)
Past medical history/comorbidities	
Insomnia	37 (62.7%)
Restless Leg Syndrome	12 (20.3%)
Depression	37 (62.7%)
Anxiety	33 (55.9%)
Other psychiatric condition	15 (26.3%)
Centrally acting medication	39 (66.1%)
Diabetes Mellitus	16 (27.1%)
Urinary incontinence	14 (23.7%)
Benign prostate hyperplasia	14 (23.7%)
Diuretic medication	9 (15.3%)
Velum (DISE)	
No collapse	2 (4.4%)
Partial anterior-posterior	17 (37.0%)
Complete anterior-posterior	22 (47.8%)
Partial concentric	5 (10.9%)
Oropharynx (DISE)	
No collapse	24 (52.2%)
Partial lateral wall	16 (34.8%)
Complete lateral wall	6 (13.0%)
Tongue Base (DISE)	
No collapse	16 (34.8%)
Partial anterior-posterior	22 (47.8%)
Complete anterior-posterior	8 (17.4%)
Epiglottis (DISE)	
No collapse	14 (30.4%)
Partial anterior-posterior	21 (45.7%)
Complete anterior-posterior	11 (23.9%)
Pre-operative sleep study data	
Apnea-hypopnea index (AHI)	38.7 (16.8)
Central apnea index (CI)	2.2 (2.9)
O2 nadir	80.6 (6.1)
Average O2 saturation	93.8 (1.7)
Percentage time O2 saturation < 88%	4.3 (7.9)
Total sleep time (min)	353 (64)
Sleep onset latency (min)	18.3 (17.6)
Wake after sleep onset (min)	64.6 (28.1)
Sleep efficiency (%)	80.1 (10.3)
Arousals per hour	36.2 (189)
Pre-op Epworth Sleepiness Scale	10.7 (9.6)
Voltage (V) at activation	1.45 (0.63)
Voltage (V) at 9 months	1.98 (0.76)

Differences in patient characteristics and HGNS use outcomes between clusters were compared using two-sided t-tests and Fisher’s exact tests. We performed a post-hoc power analysis to estimate the power of our sample to detect differences in the primary outcome (adherence rate) from 0 to 3 months to 6–9 months. Statistical significance was set at *p* < 0.05 for all analyses. SAS software version 9.4 (Cary, NC) was used for all analyses. Python version 3.11 was used for all machine learning programming, including clustering analysis.

## Results

A total of 59 patients with HGNS use data through 9 months post-activation were included. The mean (SD) age at time of HGNS implantation was 62.7 (11.2) years, and the mean (SD) BMI was 28.5 kg/m^2^ (3.2). The majority of patients were male (78%) and White (98.3%). Comorbid depression, anxiety, and insomnia were present in over half of the sample (62.7%, 55.9%, and 62.7%, respectively). The mean (SD) preoperative AHI was 38.7 events/hour (16.8) and range was 15.1 events/hour to 90.7 events/hour. The mean (SD) oxygen nadir was 80.6 (6.1). Baseline characteristics are summarized in Table 1. Among the 37 patients with a reported history of insomnia, 15 (40.5%) had a documented insomnia severity scale score, and the mean (SD) score was 15.3 (5.6). Among the comorbidities listed in Tables 1 and 7 patients (11.9%) had no comorbidities, 5 patients (8.5%) had one comorbidity, and 47 patients (79.7%) had more than one comorbidity.

The mean (SD) device voltage at activation, 3 months, 6 months, and 9 months was 1.45 V (0.63), 2.05 V (0.69), 2.06 V (0.70), and 1.98 V (0.76), respectively. Other device settings at activation and follow-up are detailed in Table 2. Most patients were activated with the +/-/+ (*n* = 28, 47.5%) or off/-/off (*n* = 26, 44.1%) electrode configuration. There were two patients who underwent a change in their electrode configuration of the course of the observation period. Post-operative outcomes including ESS, AHI, oxygen nadir, and surgical success by Sher_20_ criteria are provided in Table 3. The mean (SD) time to most recent ESS after activation was 19.7 (13.0) months and the mean (SD) time to the most recent sleep study after activation was 19.0 (13.6) months. A total of 16 patients (35.6%) achieved success by Sher20 criteria.

**Table 2:** Device settings

	Activation	9 months
Electrode configuration		
A (+/-/+)	28 (47.5%)	26 (44.1%)
B (off/-/off)	26 (44.1%)	27 (45.8%)
C (-/off/-)	5 (8.5%)	5 (8.5%)
D (-/-/-)	0	1 (1.7%)
E (-/+/-)	0	0
Pulse width (microseconds)		
60	38 (64.4%)	38 (64.4%)
90	20 (33.9%)	20 (33.9%)
120	1 (1.7%)	1 (1.7%)
Frequency (Hz)		
20	2 (3.4%)	2 (3.4%)
25	3 (5.1%)	3 (5.1%)
30	4 (6.8%)	4 (6.8%)
33	49 (83.1%)	49 (83.1%)
40	1 (1.7%)	1 (1.7%)
Duration (Hours), mean (SD)	8.7 (1.1)	8.7 (1.1)
Pause time (Minutes), mean (SD)	21.9 (7.0)	21.9 (7.0)
Start delay (Minutes), mean (SD)	39.6 (14.0)	39.7 (13.9)

**Table 3:** Subjective and objective sleep outcomes

	Preoperative (mean, SD)	Postoperative (mean, SD)	Mean difference (95% CI)	*p*-value
AHI (events/hr)*	39.3 (17.1)	25.5 (18.4)	-14.3 (-20.1 to -8.5)	< 0.001
O2 nadir*	80.6 (6.2)	82.6 (7.7)	1.93 (-0.53 to 4.40)	0.121
ESS**	10.0 (5.7)	6.3 (5.5)	-3.63 (-5.3 to -2.0)	< 0.001

*Pre-and Post-operative sleep studies available for 48 patients; 12 of the 48 post-operative sleep studies (24.5%) were titration studies

**Pre- and Post-ESS scores both available for 38 patients

The percentage of nights used, percentage of nights used for at least 4 h, and time used per night decreased significantly across all time points (Table 4). Pauses per night increased over the first 9 months (*p* = 0.008). The number (%) of patients using their device for at least 4 h per night was 56 (94.9%), 55 (93.2%), and 50 (84.8%) at 0–3, 3–6, and 6–9 months, respectively (*p* = 0.034 for change over time). When using the adherence definition of 4 h/night for 70% of nights, the adherence rate decreased from 79.7% at 0–3 months to 59.3% at 3–6 months to 52.5% at 6–9 months (*p* < 0.001). Based on a post-hoc power analysis with a sample size of 59 and alpha of 0.05, the estimated power to detect differences in adherence rate across the study time period was 88.7%. There were no sociodemographic, anthropometric, or clinical predictors of adherence at 6 to 9 months based on logistic regression models (Table 5).

**Table 4:** HGNS use outcomes over time

	0 to 3 months	3 to 6 months	6 to 9 months	*p*-value
Percentage of nights used (mean, SD)	91.7 (12.5)	85.9 (18.7)	81.1 (23.5)	< 0.001
Percentage of nights used for at least 4 h (mean, SD)	81.4 (20.5)	71.0 (24.5)	65.3 (28.2)	< 0.001
Time used per night, minutes (mean, SD)	425 (106)	388 (113)	362 (115)	< 0.001
Pauses per night (mean, SD)	0.52 (0.67)	0.81 (1.1)	0.96 (1.4)	0.008
Number (%) using at least 4 h per night	56 (94.9%)	55 (93.2%)	50 (84.8%)	0.034
Number (%) using at least 4 h for 70% of nights	47 (79.7%)	35 (59.3%)	31 (52.5%)	< 0.001

**Table 5:** Logistic models for predictors of HGNS adherence 6–9 months

	Odds ratio and 95% confidence interval	*p*-value
Age (mean, SD); per 5-year increase	1.24 (0.97 to 1.59)	0.088
Sex		
Female	Reference	
Male	0.25 (0.06 to 1.04)	0.056
Median household income (neighborhood level); per 10,000 increase	0.98 (0.84 to 1.13)	0.739
Body mass index	1.05 (0.90 to 1.24)	0.523
Past medical history		
Insomnia	1.18 (0.41 to 3.39)	0.763
Restless Leg Syndrome	0.88 (0.25 to 3.13)	0.843
Depression	0.88 (0.31 to 2.54)	0.813
Anxiety	0.91 (0.33 to 2.55)	0.859
Other psychiatric condition	0.72 (0.22 to 2.36)	0.591
Centrally acting medication	1.17 (0.40 to 3.43)	0.779
Diabetes Mellitus	2.53 (0.75 to 8.53)	0.134
Urinary incontinence	2.86 (0.78 to 10.5)	0.113
Benign prostate hyperplasia	0.88 (0.25 to 2.91)	0.827
Diuretic medication	0.68 (0.16 to 2.84)	0.599
Velum (DISE)		
No collapse	n/a	
Partial anterior-posterior	Reference	
Complete anterior-posterior	1.07 (0.3 to 3.80)	0.921
Partial concentric	0.22 (0.02 to 2.42)	0.217
Oropharynx (DISE)		
No collapse	Reference	
Partial lateral wall	0.43 (0.12 to 1.57)	0.201
Complete lateral wall	1.43 (0.22 to 9.37)	0.710
Tongue Base (DISE)		
No collapse	Reference	
Partial anterior-posterior	1.00 (0.28 to 3.63)	1.000
Complete anterior-posterior	1.67 (0.29 to 9.45)	0.564
Epiglottis (DISE)		
No collapse	Reference	
Partial anterior-posterior	1.10 (0.28 to 4.26)	0.890
Complete anterior-posterior	1.20 (0.25 to 5.84)	0.822
Pre-operative sleep study data		
Apnea-hypopnea index (AHI)	1.01 (0.98 to 1.05)	0.534
Central apnea index (CI)	1.07 (0.86 to 1.34)	0.536
O2 nadir	0.99 (0.91 to 1.09)	0.889
Average O2 saturation	0.88 (0.62 to 1.25)	0.473
Percentage time O2 saturation < 88%	0.99 (0.92 to 1.07)	0.879
Total sleep time (min)	1.00 (0.99 to 1.01)	0.939
Sleep onset latency (min)	1.03 (0.98 to 1.08)	0.217
Wake after sleep onset (min)	1.00 (0.97 to 1.02)	0.914
Sleep efficiency (%)	0.98 (0.92 to 1.05)	0.567
Arousals per hour	0.99 (0.95 to 1.03)	0.588
Pre-op Epworth Sleepiness Scale	1.02 (0.95 to 1.08)	0.630
Voltage at activation	1.05 (0.47 to 2.39)	0.893
Voltage at 9 months	1.93 (0.94 to 3.98)	0.075

Cluster analysis resulted in 2 distinct patient clusters (Supplemental File 1). Patients in cluster 0 had a significantly higher neighborhood-level household income compared to patients in cluster 1 [mean (SD) $133,103 (33,284) vs. $75,006 (17,584); *p* < 0.001)]. Patients in cluster 0 also had a longer sleep onset latency than patients in cluster 0 [mean (SD) 26.4 (20.8) minutes vs. 12.9 (13.0) minutes; *p* = 0.024)] based on the pre-operative sleep study. There were no other significant differences in baseline characteristics between the two clusters (Table 6). These results also indicated that none of the other baseline characteristics were significant in determining which cluster a particular patient belongs to.

**Table 6:** Differences in patient characteristics by cluster

	Cluster 0*(*n* = 17)	Cluster 1*(*n* = 42)	*p*-value
Age (mean, SD)	60.5 (12.0)	63.6 (10.8)	0.324
Sex			1.000
Female	4 (23.5%)	9 (21.4%)	
Male	13 (76.5%)	33 (78.6%)	
Race			1.000
Black	0 (0%)	1 (2.4%)	
White	17 (100%)	41 (97.6%)	
Neighborhood-level household income (mean, SD)	133,103 (33,284)	75,006 (17,584)	**< 0.001**
Body mass index	28.2 (4.1)	28.7 (2.9)	0.618
Past medical history			
Insomnia	11 (64.7%)	26 (61.9%)	1.000
Restless Leg Syndrome	1 (5.9%)	11 (26.2%)	0.150
Depression	14 (82.4%)	23 (54.8%)	0.074
Anxiety	9 (52.9%)	24 (57.1%)	0.781
Other psychiatric condition	4 (25.0%)	11 (26.8%)	1.000
Centrally acting medication	14 (82.4%)	25 (59.5%)	0.132
Diabetes Mellitus	4 (23.5%)	12 (28.5%)	0.759
Urinary incontinence	4 (23.5%)	10 (23.8%)	1.000
Benign prostate hyperplasia	3 (17.7%)	11 (26.2%)	0.737
Diuretic medication	3 (17.7%)	6 (14.3%)	0.708
Velum (DISE)			0.539
No collapse	1 (7.7%)	1 (3.0%)	
Partial anterior-posterior	3 (23.1%)	14 (42.4%)	
Complete anterior-posterior	7 (53.9%)	15 (45.5%)	
Partial concentric	2 (15.4%)	3 (9.1%)	
Oropharynx (DISE)			0.201
No collapse	9 (69.2%)	15 (45.5%)	
Partial lateral wall	2 (15.4%)	14 (42.4%)	
Complete lateral wall	2 (15.4%)	4 (12.1%)	
Tongue Base (DISE)			0.687
No collapse	5 (38.5%)	11 (33.3%)	
Partial anterior-posterior	7 (53.9%)	15 (45.5%)	
Complete anterior-posterior	1 (7.7%)	7 (21.2%)	
Epiglottis (DISE)			0.843
No collapse	4 (30.8%)	10 (30.3%)	
Partial anterior-posterior	5 (38.5%)	16 (48.5%)	
Complete anterior-posterior	4 (30.8%)	7 (21.2%)	
Pre-operative sleep study data			
Apnea-hypopnea index (AHI)	42.0 (12.4)	37.2 (18.4)	0.355
Central apnea index (CI)	1.5 (1.8)	2.6 (3.3)	0.232
O2 nadir	79.0 (6.4)	81.3 (5.9)	0.207
Average O2 saturation	94.3 (1.9)	93.4 (1.5)	0.108
Percentage time O2 saturation < 88%	3.7 (4.4)	4.6 (9.4)	0.713
Total sleep time (min)	357 (46.8)	351 (74.0)	0.771
Sleep onset latency (min)	26.4 (20.8)	12.9 (13.0)	**0.024**
Wake after sleep onset (min)	65.7 (30.1)	63.9 (27.5)	0.863
Sleep efficiency (%)	79.7 (9.2)	80.4 (11.1)	0.835
Arousals per hour	41.4 (24.9)	33.1 (14.1)	0.237
Pre-op Epworth Sleepiness Scale	11.3 (4.4)	10.4 (11.4)	0.788
Voltage at activation	1.39 (0.60)	1.47 (0.65)	0.665
Voltage at 9 months	1.94 (0.74)	2.00 (0.78)	0.754

*Clusters were determined using a Gaussian mixture model incorporating all variables from Table 1 except for HGNS voltage

Patients in cluster 0 had a slightly higher mean percentage of nights used at 0–3 months compared to cluster 1 (95.4% vs. 89.8%), although this did not meet the threshold for statistical significance (*p* = 0.075).There were no significant differences in any of the HGNS use metrics or adherence rate between clusters at 0 to 3 months, 3 to 6 months, or 6 to 9 months (Table 7).

**Table 7:** HGNS use outcomes by cluster

0 to 3 months			
	Cluster 0*	Cluster 1*	*p*-value
Percentage of total nights used (mean, SD)	95.4 (5.6)	89.8 (14.3)	0.075
Percentage of nights used at least 4 h (mean, SD)	86.5 (13.8)	80.0 (20.8)	0.172
Time used per night, minutes (mean, SD)	430 (94.4)	415 (99.8)	0.531
Pauses per night (mean, SD)	0.59 (0.76)	0.47 (0.58)	0.451
Number (%) using at least 4 h per night	16 (94.1%)	40 (95.2%)	1.000
Number (%) using at least 4 h for 70% of nights	21 (91.3)	42 (79.3)	0.322
**3 to 6 months**			
	Cluster 0*	Cluster 1*	**p-value**
Percentage of total nights used (mean, SD)	88.8 (12.9)	84.8 (19.7)	0.396
Percentage of nights used at least 4 h (mean, SD)	71.1 (21.7)	69.6 (24.9)	0.815
Time used per night, minutes (mean, SD)	372 (101)	384 (117)	0.691
Pauses per night (mean, SD)	1.05 (1.4)	0.69 (0.85)	0.205
Number (%) using at least 4 h per night	16 (94.1%)	39 (92.9%)	1.000
Number (%) using at least 4 h for 70% of nights	12 (57.1)	27 (58.7)	1.000
**6 to 9 months**			
	Cluster 0*	Cluster 1*	**p-value**
Percentage of total nights used (mean, SD)	83.6 (15.6)	80.0 (26.1)	0.598
Percentage of nights used at least 4 h (mean, SD)	62.7 (24.8)	66.4 (29.6)	0.652
Time used per night, minutes (mean, SD)	332 (101)	374 (119)	0.208
Pauses per night (mean, SD)	1.15 (1.6)	0.89 (1.3)	0.525
Number (%) using at least 4 h per night	14 (82.4%)	36 (85.7%)	0.708
Number (%) using at least 4 h for 70% of nights	8 (47.1)	23 (54.8)	0.774

*Clusters were determined using a Gaussian mixture model incorporating all variables from Table 1 except for HGNS voltage

## Discussion

Our study provides novel insights into long-term HGNS use patterns and changes in device use over the first 9 months of therapy. Notably, patients used their HGNS devices for a lower percentage of nights and less time/night the further out they were from activation. Similarly, patients paused their devices more frequently over time. The adherence rate decreased over time and was 52.5% at 9 months. Although the cluster analysis did not yield any significant differences in HGNS use patterns between groups, it provides an analytic framework for future work with larger sample sizes.

The long-term HGNS use patterns identified in our cohort are supported by other studies in literature. We found that at 6 to 9 months post-HGNS activation, patients used their device on average 362 min/night (6.03 h/night), and 84.8% of patients used their device at least 4 h/night. In separate ADHERE database studies, patients were found to use their devices for an average of 5.6 h/night at 14.3 months post-implant,^12^ and 88-91% of patients used their devices for at least 4 h/night at 12 months follow-up [21]. These results are encouraging, but they are tempered by our finding that only 52.5% of patients were adherent HGNS therapy at long-term follow-up when using CMS PAP adherence definition, which incorporates percentage of nights used for at least 4 h.

Although the CMS PAP adherence criterion is not validated for the HNGS population, it provides context for our findings and highlights several areas for future research. Future studies should report granular HGNS use data, including percentage of nights used for at least 4 h, as this will allow for more standardized comparison across studies. In a novel cluster analysis, Soose et al. identified six distinct patterns of HGNS use (e.g. variable use with frequent missing days) [22], which further illustrates the nuances in adherence data. While prior studies have identified stimulation-related discomfort as a general barrier to HGNS use [12, 23], future work should explore reasons underlying specific patterns of use, such as frequent missed days or frequent pauses. Finally, additional research is needed to better understand the relationship between HGNS use and long-term outcomes such as apnea-hypopnea index, subjective sleepiness, and cardiovascular morbidity.

Our study is one of the first to evaluate changes in HGNS use over time. The finding that mean percentage of nights used and time per night used decreased consecutively over each 3-month interval warrants further investigation. Stimulation-related discomfort has been identified as a barrier to HGNS use and could play a role in these findings. Pre-operative sleepiness severity was identified as a predictor of HGNS use by one study [24], but our study and others have found no significant correlation; [25, 26] this is an area for further investigation. Literature on PAP adherence in patients with OSA has demonstrated similar decreases in PAP use over time [27–29], and psychosocial factors such as disease risk perception, treatment outcome expectations, and social support have been found to play a key role [30, 31]. One study found that a music-based intervention to support habit formation led to improved CPAP adherence at 1-month [32]. It is plausible that factors associated poor PAP adherence could also affect HGNS use, especially when considering that HGNS recipients are population who did not tolerate or adhere to PAP.

Our cluster analysis incorporated numerous sociodemographic and clinical variables that have been associated with HGNS use in prior literature, but only two variables were differentiated between the clusters (household income and sleep onset latency) and there was no correlation with HGNS use. In k-means clustering, the goal is to arrive at clusters that contain data points that are most similar to other data points within the same cluster; for example, data points in cluster A would share similar properties, distinct from data points in cluster B. Once clusters are formed, an analysis can determine what properties are shared by data points in each cluster. Our k-means analysis did not find significant similar properties among data points belonging to each cluster. The lack of statistically significant findings may be secondary to false negatives in the setting of small sample size or a failure to capture other variables that are potentially related to HGNS use.

Other studies have identified post-operative variables that may correct with HGNS adherence, such as therapy response by Sher criteria (> 50% reduction in AHI, and post-operative AHI < 20 events/hour)^20,21^ and device stimulation amplitude [26]. Post-operative variables were not included in our regression models or cluster analysis because our aim was to identify pre-operative factors that could inform patient selection for HGNS. Despite this, post-operative sleep outcomes and stimulation amplitudes can help contextualize the adherence data. Notably, patients in our sample had a relatively lower mean reduction in AHI (-14.3 events/hour) and success rate by Sher_20_ criteria (35.6%) compared to large scale studies using the ADHERE database [12]. Given that adherence has been found to be lower among non-responders [21], our data might underestimate the overall adherence rate among all HGNS recipients.

Higher stimulation amplitude has been associated with lower device use [25, 26], which could plausibly be due to stimulation-related discomfort. Literature on HGNS stimulation amplitude is limited and varies considerably across studies, making it challenging to hypothesize how our findings regarding changes in mean stimulation amplitude (1.45 V to 1.98 V over 9 months) could impact device adherence. For example, Soose et al. report that patients increased their voltage on average 0.48 V over the first 90 days after activation [22]. Cai et al. report an average increase in voltage from 0.7 V to 1.8 V over the first four months after activation, and among patients who achieved therapeutic amplitude by four months, the average amplitude was 1.6 V [25]. Other studies have reported mean stimulation amplitudes as high as 2.15 V and 2.31 V, respectively, across the follow-up periods [24, 26]. Future research might incorporate a machine learning approach using a larger sample size and post-operative outcomes to gain a better understanding of predictors of HGNS use.

Our study has several limitations. Patients who stopped using their devices and were lost to follow-up before 9 months after activation may not have been captured in our study, which could lead to selection bias and an over-estimation of the adherence rate. Future studies should aim to include device adherence data through at least 12 months of follow-up to facilitate comparisons with other studies in literature. Additionally, our sample was from a single institution in the Midwest of the United States and may not be generalizable to all patients who undergo HGNS implantation. For example, compared to a large study using the ADHERE database [12], our sample had a similar mean age (62.7 years vs. 60.1 years) and BMI (28.5 kg/m2 vs. 29.3 kg/m2) but an overrepresentation of White patients (98.3% vs. 94.9%) and males (78% vs. 73.2%). Furthermore, there are potential unmeasured confounders not captured in our study such as presence of a bed partner or subjective stimulation-related discomfort. Finally, our relatively small sample size may have limited our ability to detect significant patterns in the cluster analysis. Despite these limitations, our study provides novel insight into the changes in HGNS use over time and highlights critical areas for further investigation.

## Electronic supplementary material

Below is the link to the electronic supplementary material.

Supplementary Material 1

## Data Availability

The data for this study are not openly available due to reasons of sensitivity but may be made available from the corresponding author upon reasonable request.
