# Supplementary material for: Evaluating changes in hypoglossal nerve stimulator use over time and long-term adherence
Source: Sleep Breath. 2025 Jul 18;29(4):249. doi: 10.1007/s11325-025-03415-y (PMC12274142; doi:10.1007/s11325-025-03415-y)

Grouping 1 (comprehensive):

Age, sex, race, household income, body mass index, insomnia, restless leg syndrome, depression, anxiety, other psychiatric or mood disorder, centrally acting medication, diabetes mellitus, urinary incontinence, history of prostate cancer or benign prostate hyperplasia, diuretic medication, pre-operative sleep study metrics (apnea index, hypopnea index, central apnea index, apnea-hypopnea index (3% rule), oxygen nadir, average oxygen saturation, percentage of time spent with oxygen saturation < 88%, total sleep time, sleep onset latency, wake after sleep onset, sleep efficiency, arousals per hour), pre-operative Epworth Sleepiness Scale score, DISE findings (velum, oropharynx, tongue base, epiglottis).

Grouping 2 (sleep and endoscopy parameters):

Apnea-hypopnea index, DISE findings (velum, oropharynx, tongue base, epiglottis)

Grouping 3 (a prior characteristics):

Age, body mass index, apnea-hypopnea index, anxiety, restless leg syndrome, insomnia

We used three different metrics to evaluate overall clustering results between the 3 groupings of variables:

1. **Dunn Index:** This measure evaluates how “good” a cluster is formed. It evaluates both compactness and little variance within a cluster, and good separation from other clusters. A higher value indicates a better clustering algorithm, and if this index drops when a column is removed, it indicates that the column was contributing to the overall quality of the cluster.
2. **Pseudo F Score:** This score represents the ratio of between-cluster variance to inter-cluster variance. A higher value is desired.
3. **Silhouette Score:** This score evaluates the quality of clustering. The value ranges from –1 to 1, with a number closer to one meaning the clusters are well-separated, and a number closer to –1 means clusters are not well-representative of the dataset.

Table S1: Cluster analysis for three column sets

|  | **Silhouette Score** | **Pseudo F Score** | **Dunn Index** |
| --- | --- | --- | --- |
| **Grouping 1 (comprehensive)** | .4969 | 79.56 | .0226 |
| **Grouping 2 (sleep parameters)** | .1249 | 9.570 | .0268 |
| **Grouping 2 (literature review)** | -.0583 | .2668 | .0204 |

Table S2: Dunn indices for Grouping 1 with sequential exclusion of variables

|  | **All variables** | **Excluding age, sex, or race** | **Excluding household income** | **Excluding body mass index** | **Excluding insomnia or restless leg syndrome** | **Excluding depression, anxiety, or other mood disorder** |
| --- | --- | --- | --- | --- | --- | --- |
| **Dunn Index** | .0226 | .0495 | .0144 | .0036 | .0030 | .0021 |

**Figures:**

Figure S1: Selecting the Number of Clusters using the Elbow Method


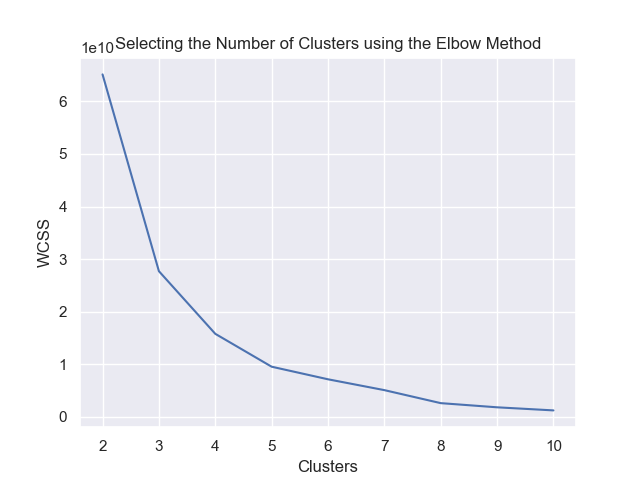


**Figure S2:** PCA Reduction Method to Visualize Clusters


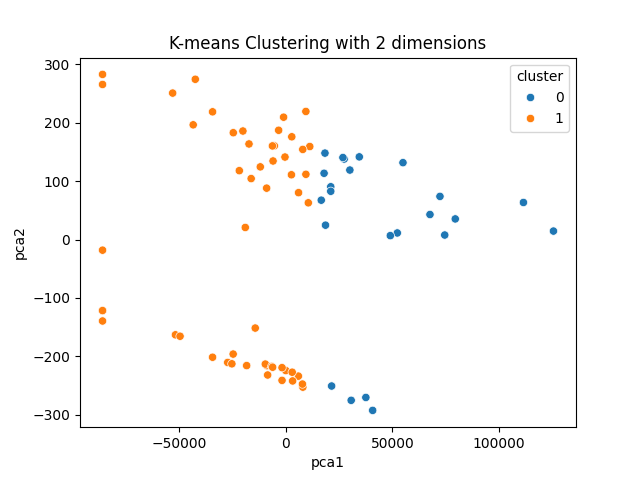

Supplement: Supplementary file 1 — Supplementary Material 1 [file 11325_2025_3415_MOESM1_ESM.docx]
